# Which antidepressant agent can be used in patients receiving tamoxifen?

**DOI:** 10.1192/j.eurpsy.2021.1156

**Published:** 2021-08-13

**Authors:** A. Maamri, H. Ghabi, H. Zalila

**Affiliations:** 1 Faculty Of Medincine Of Tunis, RAZI HOSPITAL, TUNIS, Tunisia; 2 Psychiatry, Outpatient Service, Razi Hospital, Manouba, Tunisia; 3 Outpatients Ward Of Psychiatry, Razi hospital, Manouba, Tunisia

**Keywords:** tamoxifen, antidepressant agent, Depression

## Abstract

**Introduction:**

Depression is frequently observed in carcinology. Many patients with breast cancer, receiving Tamoxifen, may need antidepressants to treat depression. Tamoxifen is a synthetic non-steroidal antioestrogen metabolized by the cytochrome P450 2D6(CYP2D6) to endoxifen which is the active metabolite of this drug. It was reported that the concomitant prescription of Tamoxifen and some antidepressant agents such as paroxetine and fluoxetine may decrease the anticancer effect of tamoxifen as they may inhibit the CYP2D6 pathway.

**Objectives:**

The objective of this case was to highlight the particularity of management of depression in patients under tamoxifen.

**Methods:**

Case report description of a patient treated with Tamoxifen for her breast cancer and who was admitted for major depression, followed by a literature review.

**Results:**

A 36 –year- old woman, had breast cancer and she underwent a mastectomy followed by chemotherapy. Since September 2016, she received 20 mg per day of Tamoxifen as an antihormonal treatment. In November 2018, she was referred to our psychiatry department for depressive symptoms. The patient was sad, she reported social withdrawal, insomnia, anhedonia, and low self-esteem. She had no history of mania or hypomania. A major depressive episode was diagnosed. We prescribed Escitalopram 10 mg per day with clinical improvement. The psychiatric and oncologic status of the patient was stable after two years under tamoxifen and Escitalopram.

**Conclusions:**

The choice of the adequate antidepressant agent in patients under Tamoxifen remains a challenge and requires a thorough knowledge of drug interactions.

